# Esketamine for postoperative sleep disturbance: clinical evidence, mechanisms, and future directions

**DOI:** 10.3389/fpsyt.2025.1612230

**Published:** 2025-11-05

**Authors:** Qijing Liu, Ying Liu, Qian Fu, Boxiong Gao, Yatao Liu

**Affiliations:** ^1^ The First School of Clinical Medicine, Lanzhou University, Lanzhou, Gansu, China; ^2^ Department of Anesthesiology and Surgery, First Hospital of Lanzhou University, Lanzhou, Gansu, China

**Keywords:** postoperative sleep disturbance, sleep quality, esketamine, ketamine, NMDA receptor antagonist

## Abstract

Postoperative sleep disturbance (PSD) is a common complication following surgery. Numerous factors can contribute to PSD, including personal factors, intraoperative factors, postoperative complications and environmental factors. PSD can lead to a range of adverse outcomes, severely impairing patients’ postoperative recovery and long-term prognosis. Esketamine, a non-competitive N-methyl-D-aspartate (NMDA) receptor antagonist and the dextrorotatory isomer of ketamine, which has stronger receptor affinity, more significant analgesic effects and better safety than ketamine. In recent years, in addition to the proven sedative, analgesic and antidepressant properties, emerging evidence highlights that esketamine may improve PSD through a variety of mechanisms, but the existing research results are still controversial. This article reviews the latest research progress of esketamine in improving PSD, and discusses its clinical efficacy and potential mechanism of action, in order to provide theoretical basis and practical guidance for optimizing perioperative anesthesia management and promoting postoperative rehabilitation of patients.

## 1 Introduction

Adequate sleep is essential for sustaining various physiological processes, particularly for preserving optimal brain function. Normal sleep pattern is typically categorized into two primary states: non-rapid eye movement (NREM) and rapid eye movement (REM) sleep. The NREM phase is further subdivided into three distinct stages, namely N1, N2, and N3, which account for approximately 5%-10%, 45%-55%, and 15%-25% of the total sleep duration in adults, respectively (1, 2). Notably, the N3 stage is characterized by delta wave activity and is frequently termed slow-wave sleep (SWS) or deep sleep. During the N3 stage, the body’s immune function is enhanced, and cognitive function is supported by clearing waste from the brain (such as β-amyloid (Aβ)) (1, 3). In adults, the REM stage typically represents approximately 20%-25% of the overall sleep duration. REM sleep is involved in the development of the nervous system and the establishment of synaptic connections, and it plays a crucial role in memory consolidation and emotion regulation (4, 5).

Normal sleep consists of multiple periodic cycles, each of about 90 minutes. A typical sleep cycle progresses in sequence through the following stages: N1→N2→N3→N2→REM sleep (2). The aging process significantly influences sleep architecture, characterized by a reduction in total sleep duration, diminished proportions of N3 and REM stages, increased sleep onset latency, higher frequency of nocturnal awakenings, and increased duration of N1 and N2 stages (6). Therefore, elderly patients are more likely to experience sleep problems.

Postoperative sleep disturbance (PSD) is a common complication among patients who undergo surgical procedures. The clinical manifestations of PSD are highly diverse and typically include difficulty falling asleep, early awakenings, increased frequency of awakenings, abnormal dream experience, daytime fatigue and other objective symptoms. Polysomnography (PSG) monitoring showed characteristic changes: sleep fragmentation, reduced total sleep time, and a significant decrease or even complete absence of SWS and REM sleep, particularly on the first night after surgery (7). It is worth noting that PSD is not merely a manifestation of postoperative sleep-wake cycle disruption, but also an important clinical indication of postoperative brain dysfunction. Therefore, it is essential to improve the PSD of patients.

The prevention and treatment of PSD are multifaceted, encompassing both non-pharmacological and pharmacological interventions. Non-pharmacological strategies primarily include environmental optimization (eg, the use of eye masks and earplugs to reduce light and noise interference), psychological and behavioral therapies (eg, relaxation training and music therapy), and traditional Chinese medicine (eg, acupoint stimulation) (8–10). Pharmacological interventions are important for improving PSD, with commonly used medications including melatonin (regulating circadian rhythm), zolpidem (short-acting sedative hypnotic), and dexmedetomidine (selective α_2_ adrenergic receptor agonist) (11–13). Recently, emerging evidence has highlighted the potential benefits of perioperative ketamine and its dextrorotatory isomer, esketamine, in reducing the incidence of PSD and improving postoperative sleep quality (14, 15). Ketamine, especially esketamine, has become the new focus of PSD intervention research due to its multiple targets of action, which can exert analgesic, antidepressant, anti-inflammatory effects and regulation of circadian rhythm.

Esketamine, as a dextrorotatory isomer of ketamine, has stronger receptor affinity, more significant analgesic effects and better safety than ketamine. This study was conducted as a narrative review of the existing literature, aiming to evaluate the efficacy of esketamine in treating PSD and explore its potential underlying mechanisms, in order to provide theoretical basis for clinical anesthesia management and postoperative rehabilitation. Given the rapid expansion of esketamine research in recent years, we employed a systematic approach to identify and select relevant publications. The detailed methodology content is illustrated in **Supplementary File S1.docx**.

## 2 Risk factors and adverse outcomes of postoperative sleep disturbance

The occurrence of PSD involves multiple factors, mainly including: (1) Patient factors: gender, age and other demographic characteristics, preoperative anxiety, depression and pre-existing sleep problems; (2) Intraoperative factors: degree of surgical trauma, intensity of stress response, type and duration of anesthesia; (3) Postoperative complications: pain, postoperative nausea and vomiting (PONV) and ward environment (noise/light interference) (2, 7, 15, 16). These multifactorial etiologies contribute to the high incidence of perioperative sleep problems, with preoperative sleep disturbance affecting up to 60% of surgical patients (17) and PSD occurring in more than 70% of individuals undergoing noncardiac procedures (16).

Notably, the impact of anesthetic drugs on sleep is particularly unique. General anesthesia produces a reversible, controllable state through drug induction, including unconsciousness, amnesia, analgesia, and immobility. Contemporary neuroscience indicates that, although both general anesthesia and natural sleep involve reversible loss of consciousness, there are both overlaps and significant disparities between general anesthesia and physiological sleep in terms of neural circuits and electroencephalogram (EEG) manifestations. Natural sleep is an active, rhythmic and cyclical process dominated by circadian rhythm and homeostasis regulation, which can promote memory formation and consolidation. In contrast, general anesthetics induce unconsciousness by acting simultaneously on multiple cortical and subcortical neural circuits, partially relying on sleep-like oscillations (eg, slow-delta oscillations). Moreover, they directly inhibit cortical neurons and subcortical arousal-promoting neurons, thereby synergistically suppressing arousal. Conversely, activation of these arousal-promoting neurons can facilitate anesthesia emergence. Although some anesthetics can induce slow-delta oscillations similar to NREM sleep, each class of drugs exhibits unique dose-dependent property (18).

**Figure 1:**
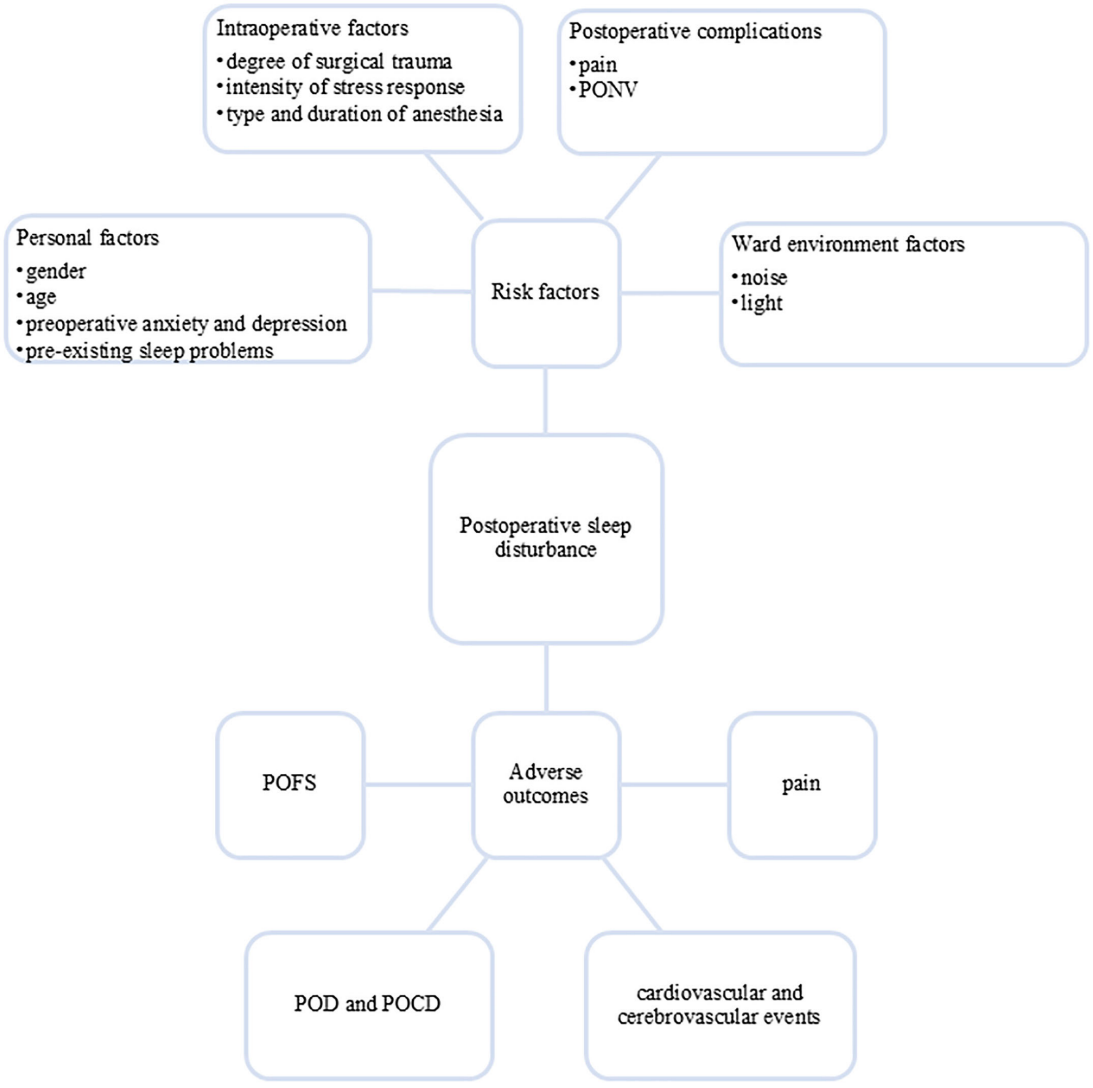
Risk factors and adverse outcomes of postoperative sleep disturbance.

PSD can cause a series of adverse outcomes, significantly impairing patients’ postoperative recovery and long-term prognosis. PSD can exacerbate postoperative pain perception (19), increase the risk of cardiovascular and cerebrovascular events (20–22), and is also a risk factor for postoperative delirium (POD) and postoperative cognitive dysfunction (POCD) (23). Additionally, PSD may lead to postoperative fatigue syndrome (POFS) (24) (see **Figure 1**). These pathophysiological changes not only delay recovery but also prolong hospital stays and increase the health care burden.

## 3 Pharmacological properties of esketamine

Ketamine, a non-competitive N-methyl-D-aspartate (NMDA) receptor antagonist, is a racemic compound comprising equal proportions of R (-)-ketamine and S (+)-ketamine enantiomers. It is primarily used as a narcotic analgesic in clinical practice. The drug exhibits unique physicochemical properties, being both water-soluble and lipid-soluble, which enable its administration via multiple routes, including intravenous, intramuscular, intraosseous, oral and intranasal pathways. Its pharmacokinetic characteristics showed that the elimination rate and distribution volume are mainly influenced by hepatic perfusion (25). Notably, Berman et al. firstly identified that ketamine also has significant antidepressant effects (26), a finding that has greatly expanded its clinical application prospects. Nevertheless, the clinical application of ketamine is constrained by its propensity to induce psychotomimetic adverse effects, such as hallucination, nightmare and restlessness.

**Figure 2:**
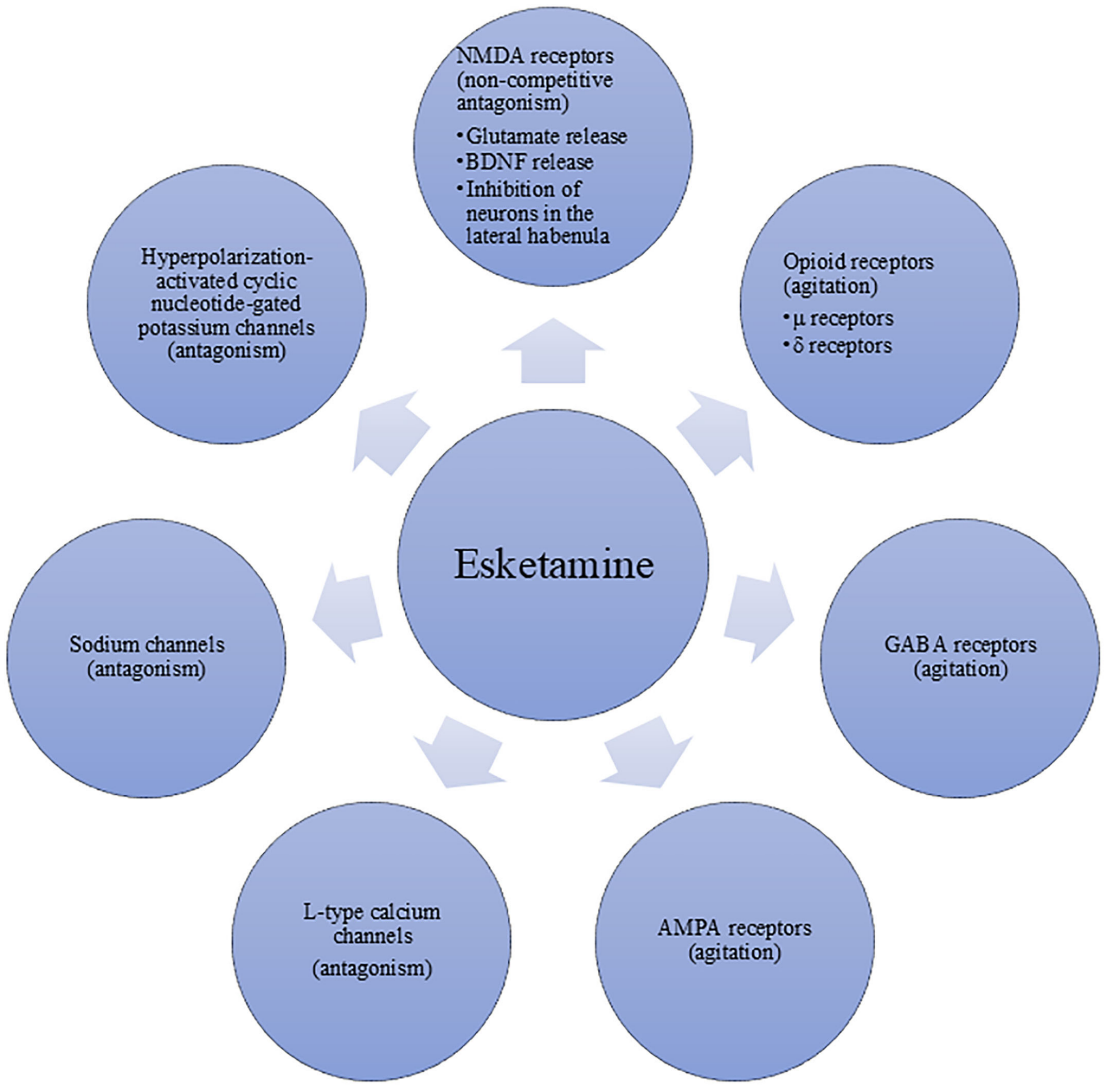
Targets of action of esketamine.

Esketamine, the S (+)-enantiomer of racemic ketamine, exerts its pharmacological effects through multiple molecular targets (see **Figure 2**). The optimal administration method of esketamine for anesthesia and analgesia is intravenous injection, the effect is dose-dependent (27). And the main mechanism by which it produces anesthetic and analgesic effects is its non-competitive antagonism of NMDA receptors. Although esketamine shares similar pharmacodynamic characteristics with ketamine, it exhibits approximately twice the binding affinity for the NMDA receptors compared to the racemate. Consequently, esketamine requires only half the dose of ketamine to achieve comparable anesthetic and analgesic effects.

Currently, the product labeling for esketamine still refers to the pharmacokinetic data of racemic ketamine. However, a clinical study conducted in Chinese patients undergoing painless gastroscopy demonstrated that the onset time and the duration of action did not differ significantly between esketamine and ketamine, but the total dose of esketamine required to reach the specified blood concentration is only 65% of ketamine. The mean elimination half-life of esketamine was approximately 4 hours. Notably, esketamine displayed higher clearance rate and shorter recovery time compared to ketamine. Esketamine is metabolized primarily by hepatic microsomal enzymes, yielding S-norketamine as its major active metabolite, which has a mean elimination half-life of approximately 6–10 hours (28). An important pharmacological characteristic of esketamine is that the plasma concentration required for its analgesic effect is significantly lower than that needed for the loss of consciousness (29, 30). This implies that even after the anesthetic effect subsides and the patients regain consciousness, the analgesic effect may still persist for a certain period. This is highly beneficial for postoperative analgesia, as it can provide a certain degree of continuous analgesic effect and reduce the need for other analgesic medications. Furthermore, it may lead to subsequent effects such as potential neuroprotection and antidepressant effects (31).

Although esketamine offers advantages such as stronger affinity, higher clearance rate, and shorter recovery time compared to ketamine, the potential adverse effects after esketamine administration warrant consideration, especially the psychomimetic symptoms. According to a literature review covering the period from 1980 to 2022, esketamine is not devoid of psychomimetic side effects. Esketamine is also associated with dose-dependent psychomimetic adverse effects, such as hallucinations, thought disorganization, depersonalization, derealization, and abnormal dreams. Even at subanesthetic (≤0.5 mg/kg) or low doses (≤0.2 mg/kg), these neuropsychiatric manifestations may occur, though they are generally mild and transient. At higher doses, sedation gradually occurs until loss of consciousness. Compared with racemic ketamine, the psychomimetic effects of esketamine are milder, and the cognitive impairment during recovery is less severe and milder (32).

The mechanism underlying the psychotomimetic adverse effects of esketamine stems from its core pharmacological action as a NMDA receptor antagonist. By blocking NMDA receptors on γ-aminobutyric acid (GABA) inhibitory interneurons, esketamine suppresses the activity of these neurons, leading to the disinhibition and abnormal excitation of downstream glutamatergic pyramidal neurons. This results in a sharp increase in glutamatergic signaling in brain regions such as the prefrontal cortex (PFC) (33). This widespread excitatory disorder further disrupts the dynamic balance between large-scale brain networks, particularly causing disconnections within the default mode network (DMN) and its connections with the salience network (SN) and the executive control network (ECN). This is closely related to abnormal self-perception and the sense of reality disintegration (34). Concurrently, esketamine significantly interferes with the function of the temporoparietal junction (TPJ), a brain region responsible for integrating visual, vestibular, and proprioceptive information. Its dysfunction leads to failure in multisensory integration, directly causing symptoms such as hallucinations, perceptual distortions, and out-of-body experiences (35). Therefore, the psychotomimetic adverse effects of esketamine are not caused by a single mechanism but are the collective result of a series of cascading reactions triggered by its NMDA receptor antagonism, ultimately leading to the dysfunction of multiple brain networks.

## 4 Esketamine in clinical practice for pain

Building upon its unique pharmacological profile, esketamine has been investigated for several clinical applications beyond anesthesia. An increasing number of clinical studies have confirmed that esketamine not only provides significant analgesic effects (36), but also effectively alleviates negative emotions (31), while significantly enhancing patients’ postoperative recovery quality (37).

The foremost advantage of esketamine is its potent analgesic and opioid-sparing effects. Multiple studies have consistently demonstrated that low-dose esketamine, either as an adjuvant during anesthesia or in patient-controlled intravenous analgesia (PCIA) pumps, significantly reduces postoperative pain scores and reduces the consumption of opioids, especially in cesarean section. Moreover, some studies have shown that perioperative esketamine administration markedly decreases the incidence of PONV (38–40). This beneficial effect may be associated with diminished perioperative opioid utilization and enhanced maintenance of hemodynamic stability.

While numerous studies report significant reductions in pain scores and opioid requirements, others show neutral or negative findings (41, 42). Firstly, this discrepancy can be attributed to several factors, including varying surgical models (major abdominal or superficial surgery), divergent dosing regimens (bolus or continuous infusion), and differences in the timing of administration (pre-incision or intraoperative). Secondly, while esketamine may demonstrate benefits in acute pain control, its long-term effects on preventing chronic postsurgical pain (CPSP) remain inadequately explored (43, 44). Additionally, the concomitant use of other analgesics in multimodal strategies can also obscure its specific contribution (45). Lastly, the contribution of active metabolites like S-norketamine, with its longer half-life and distinct pharmacological activity, to the overall analgesic is often not accounted for in clinical studies (46).

While esketamine holds considerable promise as a multimodal analgesic adjuvant, its application is not without challenges. Future research must prioritize the standardization of dosing, the identification of patient subgroups most likely to benefit, and the rigorous assessment of long-term outcomes to fully define its role in enhanced recovery protocols.

## 5 Clinical evidence that esketamine improves postoperative sleep disturbance

In recent years, a number of clinical studies have investigated the effects of esketamine on postoperative sleep quality. The results are somewhat inconsistent, but generally indicate that esketamine may improve postoperative sleep quality through multiple mechanisms.

### 5.1 Effect of esketamine on subjective sleep quality

Accumulating evidence demonstrates that perioperative administration of esketamine significantly reduces the incidence of PSD and enhances patients’ self-reported sleep quality. For instance, Qiu et al. found that in patients undergoing gynecological laparoscopic surgery, intraoperative intravenous infusion of esketamine (0.3 mg/kg/h) significantly lowered the incidence of PSD on the first and third postoperative days, from 44.0% to 22.8% and from 19.8% to 7.6%, respectively (15). In another study involving laparoscopic gastric carcinoma resection, a combined regimen of intravenous esketamine (0.5 mg/kg) after induction and PCIA containing esketamine (1 mg/kg) effectively alleviated postoperative pain, enhanced sleep quality, reduced fatigue, and accelerated patient recovery (47). Research on elderly patients undergoing laparoscopic gastrointestinal tumor surgery revealed that intravenous esketamine (0.25 mg/kg) after induction, combined with continuous intraoperative infusion (0.1 mg/kg/h) improved sleep quality from postoperative day 1 through day 3 (48). Additionally, esketamine has proven effective in enhancing sleep quality for patients undergoing video-assisted thoracoscopic surgery (VATS), whether administered as a single pre-induction intravenous dose (0.5 mg/kg) or as a 24-hour postoperative infusion (1.5 mcg/ml sufentanil combined with 0.75 mcg/ml esketamine) (43, 49). It is worth noting that these studies employed subjective sleep assessment scales, which consistently demonstrating that perioperative administration of esketamine positively impacts patients’ postoperative sleep quality.

### 5.2 Effect of esketamine on sleep structure

Previous electrophysiological study in rats has shown that intraperitoneal ketamine injection selectively increases slow-wave activity (SWA) in the EEG during NREM sleep and modulates the expression of brain-derived neurotrophic factor (BDNF) in central brain regions (50). BDNF, a neuropeptide abundant in the CNS, is critical for neuroplasticity. In patients with treatment-resistant major depressive disorder (MDD), ketamine infusion has been observed to significantly influence sleep architecture, as indicated by increased total sleep duration, SWS, and REM sleep compared to the baseline. Furthermore, reductions in N1, N2, REM sleep latency, and wake time were noted on the day following ketamine administration (51). A randomized controlled trial (RCT) involving patients undergoing open abdominal gynecological surgery revealed that intraoperative intravenous infusion of esketamine (0.2 mg/kg/h) combined with an additional esketamine (50 mg) added to PCIA significantly increased the proportion of N3 sleep on the first postoperative night, from 8.9% to 15.6%. However, no significant differences were observed between the intervention and control groups in subjective sleep quality scores or other sleep architecture components, including N1, N2, and REM sleep (52).

### 5.3 Controversy

Although most studies support the positive effect of esketamine on improving postoperative sleep, some studies still yield negative results. For example, one study observed that repeated low-dose intravenous esketamine administration did not enhance sleep quality in elderly patients undergoing total hip or knee arthroplasty (53). Similarly, Sun et al. found that intraoperative esketamine administration failed to improve sleep outcomes in patients undergoing laparoscopic radical resection for colorectal cancer (54).

Existing studies have shown that the effects of perioperative esketamine on postoperative sleep quality are heterogeneous, which may be related to differences in study populations and different dosing regimens (dose/timing). Comprehensive evidence showed that the current clinical studies mainly used intravenous administration, including: intraoperative single administration (0.1-0.5 mg/kg); intraoperative continuous infusion (0.1-0.3 mg/kg/h); postoperative continuous administration (50 mg or 0.5–1 mg/kg combined with opioids). Although the mechanism has not been fully elucidated, existing evidence supports the positive significance of perioperative intravenous esketamine in improving postoperative sleep quality.

While the above clinical evidence suggests that esketamine may be a promising agent for alleviating PSD, it is imperative to acknowledge that the application of esketamine for PSD is strictly off-label. Clinicians and researchers must exercise utmost caution due to several inherent risks. First and most commonly, there are mental related adverse reactions such as nightmares, dizziness, hallucinations and mental confusion (32). Even though these symptoms are mostly temporary, we need to conduct appropriate monitoring to ensure the safety and comfort of the patients. Secondly, esketamine can cause a dose-dependent increase in blood pressure and heart rate (55), which warrants careful consideration in postoperative patients, especially those with cardiovascular instability. Thirdly, the long-term safety of repeated esketamine administration for sleep modulation, particularly in surgical populations, remains largely unexplored. Finally, as a schedule-controlled substance, esketamine carries a risk of misuse and dependence. Its use must be recorded in detail and be restricted to use in supervised medical environments.

## 6 Possible mechanisms of esketamine improving postoperative sleep quality

The lateral preoptic (LPO) hypothalamus is a core brain region that generates and maintains sleep (including NREM and REM sleep). The excitability of neurons in this brain region is highly dependent on NMDA receptors (particularly the GluN1 subunit). A experiment reported that specifically deleting NMDA receptors in the LPO leads to severe insomnia, indicating that the normal function of NMDA receptors is necessary for maintaining sleep homeostasis (56). Furthermore, recent studies have confirmed that the discharge patterns of dopaminergic neurons in the ventral tegmental area (VTA) play a crucial role in the transition between sleep and wakefulness. One of the key mechanisms of the NMDA receptor antagonist is its action on the GABAergic interneurons in the VTA. Blocking the NMDA receptors on these inhibitory neurons will relieve their inhibition on the dopaminergic neurons, resulting in a sharp increase in dopamine release (57, 58). The surge of dopamine will drive awakening (59). These give rise to a core paradox: How can NMDA receptor antagonists improve sleep?

At present, the specific mechanisms of ketamine and esketamine to improve sleep quality after surgery are unclear. The regulation of sleep-wake states relies on the balance of complex neural networks and neurotransmitter systems within the brain, in which NMDA receptors are widely involved. Some studies indicate that NMDA receptor antagonists can enhance SWA on EEG (60). Esketamine may induce or enhance the recovery of deep sleep, which is similar to physiological SWS in EEG, through its action on NMDA receptors in the cortex and thalamus. In addition, ketamine can also participate in a more extensive regulation of sleep-wake cycle homeostasis by modulating key neuropeptides in the hypothalamic endogenous sleep-wake regulatory system, such as inhibiting the release of the wake-promoting neuropeptide orexin (OX) and potentially promoting the activity of the sleep-promoting neuropeptide melanin-concentrating hormone (MCH) (61).

**Figure 3:**
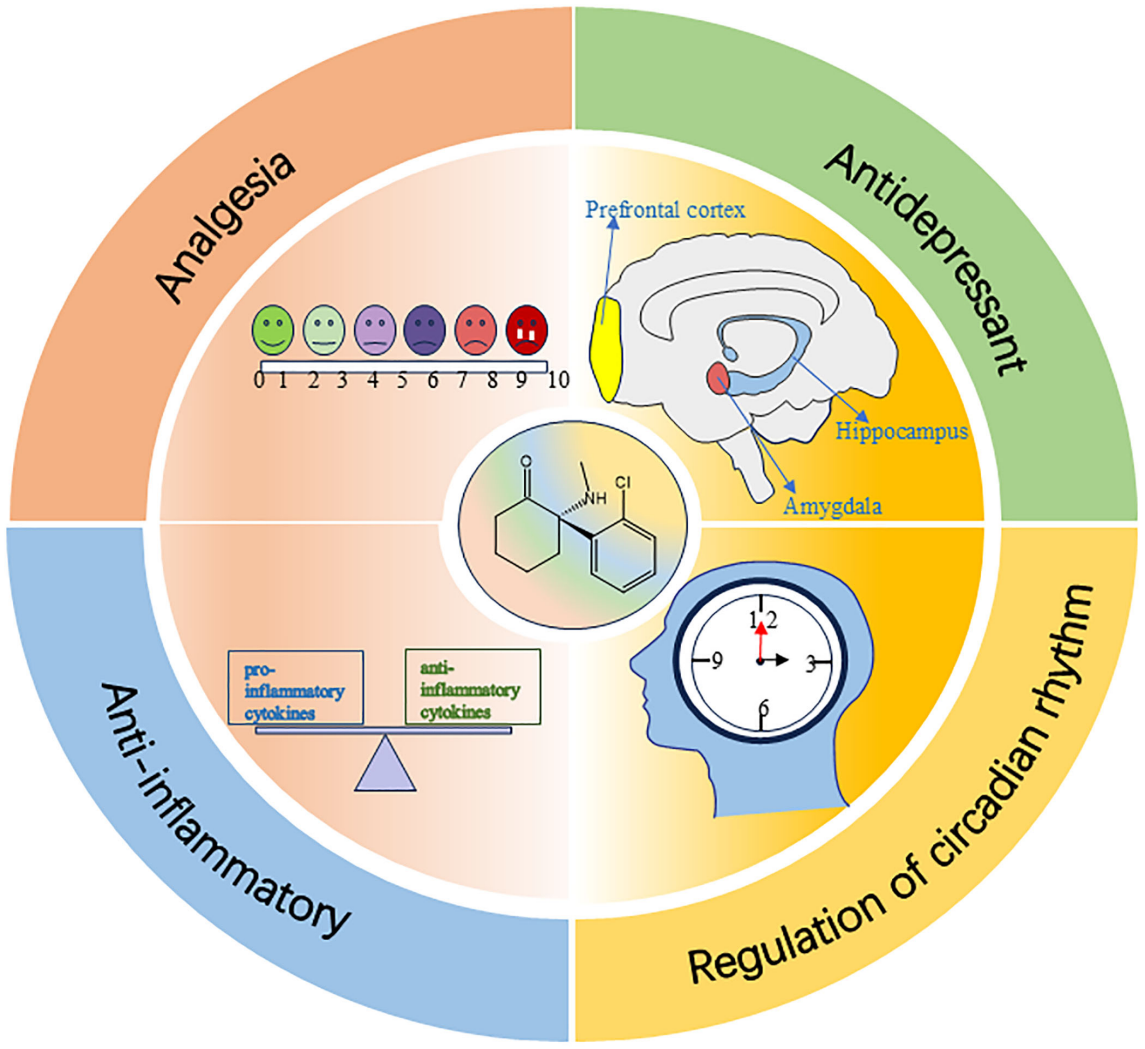
Indirect mechanisms of esketamine in improving postoperative sleep disturbance.

The effect of esketamine in improving postoperative sleep is not only achieved by directly regulating the sleep-wake center circuit, but may also result from its multiple indirect mechanisms, such as analgesia, anti-depression, anti-inflammation, and regulation of circadian rhythm (see **Figure 3**).

### 6.1 Analgesia

Patients undergoing surgical treatment often experience varying degrees of pain both before and after surgery. The bidirectional relationship between pain and sleep has been widely acknowledged: pain can disrupt sleep, resulting in prolonged sleep latency and reduced total sleep time; conversely, sleep disturbance is an important predictor of pain (62). Opioids are the most commonly used analgesic drugs. However, long-term or irregular use of opioids can produce related adverse reactions, among which sleep disturbance is one of the adverse reactions of opioid use. Sleep disturbance can lead to decreased pain tolerance and further increase the use of opioids, presenting a vicious cycle (63). In a study of intravenous morphine administration in healthy painless young adults, it was found that intravenous morphine administration at clinical doses altered sleep structure in healthy painless subjects, with decreased SWS and REM sleep, and an increase in N2 on PSG (64). A large amount of evidence indicates that esketamine provides effective analgesic effects, although several studies failed to reach statistical significance. Additionally, the study has also found that intraoperative use of esketamine not only improves postoperative sleep but also reduces postoperative movement pain scores and analgesic drug consumption (15).

The possible mechanisms of esketamine analgesia: (1) NMDA receptors are crucial excitatory glutamate receptors in the central nervous system (CNS) and play a pivotal role in the transmission of nociceptive signals (65). When a noxious stimulus occurs, the presynaptic membrane releases the excitatory neurotransmitter glutamate, which activates the NMDA receptors. This activation triggers a voltage-dependent influx of sodium ions (Na^+^) and calcium ions (Ca^2+^) and an efflux of potassium ions (K^+^), ultimately leading to pain (66). As an NMDA receptor antagonist, esketamine may attenuate glutamate-mediated nociceptive signal transmission by binding to NMDA receptors, thereby preventing glutamate from binding to its receptors and reducing the time and frequency of receptor channel opening. This mechanism helps to alleviate pain. (2) The nitric oxide (NO)/cyclic guanosine 3′,5′-monophosphate (cGMP) signaling pathway has been identified as a critical mediator in the pathogenesis of chronic pain, particularly in inflammatory pain (67). NO is a biological mediator widely involved in pain regulation. The brain is the primary source of NO in the body, and the main stimulus for NO synthesis in the brain is Ca^2+^ influx through NMDA glutamate receptors. Upon NMDA receptor activation, Ca^2+^ enters the cell and binds to calmodulin (CaM) to form a Ca^2+^-CaM complex. This complex can activate neuronal nitric oxide synthase (nNOS) to promote NO production (68). Given that esketamine acts as an NMDA receptor antagonist, it may inhibit NMDA receptor activation, thereby affecting the production of NO and inhibiting inflammatory pain. (3) In addition, esketamine also has a certain binding affinity for opioid receptors (eg, μ- and δ-opioid receptors), which may enhance its analgesic effects by regulating the activity of these receptors (69).

### 6.2 Antidepressant

Sleep disturbance has long been recognized as a core symptom of depression. However, emerging research has revealed that sleep disturbance not only co-occurs with depressive disorders but also serves as an independent risk factor for their onset. Long-term persistent sleep disturbance can significantly increase the risk of individuals suffering from mental illnesses. As one of the most common mental illnesses, depression has a complex bidirectional relationship with sleep disturbance (70). The antidepressant effects of ketamine represent a major breakthrough in the field of mental health. Unlike conventional antidepressants, ketamine exerts its therapeutic effects within just a few hours and reduces suicidal ideation in the short term (71, 72). Furthermore, clinical investigation has demonstrated ketamine’s efficacy in alleviating insomnia severity among patients with depressive disorders (73). Notably, in 2019, United States Food and Drug Administration (FDA) granted approval for esketamine as a therapeutic intervention for treatment-resistant depression (TRD) (74). As a novel antidepressant, the efficacy of esketamine has been verified across various surgical populations (31, 75–80). Recent research has shown that a single low-dose administration of esketamine (0.2 mg/kg) following childbirth can result in a 75% reduction in major depressive episodes at 42 days postpartum among women experiencing prenatal depression (81). The antidepressant mechanisms of ketamine and esketamine have garnered significant interest in recent years.

The possible antidepressant mechanisms of esketamine: (1) The lateral habenula (LHb) nucleus is a small subcortical nucleus in the brain, which inhibits the activity of reward-related dopaminergic neurons and plays a crucial role in the regulation of negative emotions. Research has shown that the burst-like firing activity of the LHb is closely linked to the development of depression, and this firing pattern is dependent on NMDA receptors. By blocking NMDA receptors, esketamine inhibits the excessive activity of the LHb and modulates its functional connectivity with brain regions associated with the reward system and cognitive control, thereby exerting its rapid antidepressant effects (82). (2) Several studies have highlighted the pivotal involvement of NMDA receptors in the underlying mechanisms of depression. NMDA receptors have multiple subtypes and are widely expressed throughout the CNS. It is notable that the expression of GluN2A and GluN2B subtypes is closely related to the occurrence of depression (83, 84). As NMDA receptor antagonists, ketamine and esketamine may exert rapid antidepressant effects by blocking these receptors. (3) The antidepressant properties of ketamine and esketamine can be explained not only by their direct antagonism of glutamatergic NMDA receptors but also through their selective blockade of NMDA receptors located on GABA inhibitory interneurons. This blockade leads to the disinhibition of pyramidal neurons, enhancing glutamate release. Subsequently, glutamate activates α-amino-3-hydroxy-5-methyl-4-isoxazolepropionic acid (AMPA) receptors and promotes the secretion of BDNF (85). It has been found that ketamine affects the level of BDNF, and this change is associated with SWS and mood regulation (51). (4) In addition, esketamine exerts antidepressant effects by promoting the activation of the mammalian target of rapamycin (mTOR) signaling pathway in the PFC, increasing synaptic plasticity (86). (5) Moreover, hydroxynorketamine (HNK), a metabolite of ketamine, is also one of the important mechanisms underlying its antidepressant effects (87). Esketamine has been found to have a 10% higher demethylation rate than ketamine (88), which may contribute to its potent antidepressant effects. The antidepressant effects of HNK do not depend on the inhibition of NMDA receptors, but are achieved by enhancing AMPA receptor function. This mechanism further supports the critical position of AMPA receptors in the antidepressant effects of ketamine and esketamine.

### 6.3 Anti-inflammatory

Surgical trauma, inflammatory response, and postoperative pain are major factors affecting postoperative sleep quality (7). Compared with patients undergoing minimally invasive procedures such as laparoscopic cholecystectomy, those who undergo major abdominal surgery experience more severe circadian rhythm disorders and worse subjective recovery parameters (89). The inflammatory response is the body’s natural defense mechanism against surgical trauma. Extensive surgical trauma or the presence of other influencing factors can trigger a systemic inflammatory response, leading to an imbalance in the level of inflammatory factors. Scientific investigation has established interconnections among inflammatory markers, sleep disruption, and depressive states (90). Specific cytokines, including tumor necrosis factor (TNF) and interleukins (91), which are involved in postoperative inflammatory processes, may contribute to the development of PSD. Experimental evidence indicates that administration of exogenous TNF or interleukin-1 (IL-1) can trigger symptoms similar to sleep deprivation (92, 93), such as excessive somnolence, fatigue, cognitive impairment, and hyperalgesia. These symptoms imply a potentially significant role of these inflammatory mediators in the pathogenesis of PSD. We hypothesized that reducing the release of inflammatory factors could improve sleep disorders and alleviate depressive symptoms. In elderly patients, anesthesia induction using propofol in combination with esketamine demonstrated superior clinical outcomes compared to propofol paired with sufentanil. This approach enhances hemodynamic stability, mitigates surgical stress and inflammatory reactions (as indicated by decreased levels of C-reactive protein (CRP), procalcitonin (PCT), and white blood cell (WBC) counts), reduces anesthesia duration, and promotes the restoration of postoperative cognitive function (94). These effects have been validated in several studies (31, 95–98), indicating that esketamine has potential clinical benefits for its anti-inflammatory effects.

The possible anti-inflammatory mechanisms of esketamine: (1) By inhibiting leukocyte activation, esketamine attenuates the generation of pro-inflammatory cytokines, including tumor necrosis factor-α (TNF-α), interleukin-6 (IL-6), and interleukin-8 (IL-8), while enhancing the release of anti-inflammatory cytokines, such as interleukin-4 (IL-4) and interleukin-10 (IL-10). This mechanism consequently reduces inflammation-induced neural damage (99). (2) Lipopolysaccharide (LPS) is an endotoxin that induces significant upregulation of inflammatory mediators, including cytokine production and enhancement of enzyme activity. This process involves several key molecular events, such as the phosphorylation of nuclear factor-κB (NF-κB) and its nuclear translocation, as well as the elevation of Ca^2+^ level and the phosphorylation of calmodulin-dependent protein kinase II (CaMK II). These molecular events interact and work together to drive the inflammatory process. Like ketamine, esketamine exhibits potential anti-inflammatory properties through antagonism of NMDA receptors, reduction of Ca^2+^ level, inhibition of CaMK II phosphorylation, and inhibition of NF-κB phosphorylation and nuclear translocation (100). (3) There is a mutually promoting relationship between oxidative stress and inflammation. As a crucial transcriptional regulator of the cellular oxidative stress response, nuclear factor erythroid 2-related factor 2 (Nrf2) modulates the expression of genes responsible for antioxidant and anti-inflammatory activities, playing a key role in safeguarding cells against the detrimental impacts of oxidative stress and inflammatory processes (101). Research has demonstrated that esketamine induces rapid antidepressant effects in adolescent mice exposed to LPS, an effect linked to the stimulation of Nrf2-mediated anti-inflammatory signaling pathways (102).

### 6.4 Regulation of circadian rhythm

Sleep regulation is governed by two primary systems: the circadian rhythm and homeostasis regulation (103). The circadian rhythm represents the 24-hour cycle of physiological and behavioral variations in organisms, controlled by the circadian clock within the suprachiasmatic nucleus (SCN) of the hypothalamus. In humans, the most prominent circadian rhythm is the alternation between sleep and wakefulness. Circadian rhythm disturbance is closely related to a variety of diseases, such as depression, sleep disorders, and metabolic syndrome. In recent years, studies have found that ketamine and esketamine not only possess analgesic and rapid antidepressant effects but also play a role in regulating circadian rhythm (104, 105).

The possible mechanisms by which esketamine regulates circadian rhythm: (1) In mammals, the core circadian clock genes include period genes (Per1, Per2, Per3), cryptochrome genes (Cry1, Cry2), Bmal genes (Bmal1, Bmal2), and Clock genes (Clock, NPAS2). The Clock: Bmal1 complex serves as a central transcription factor in the mammalian circadian clock, modulating the expression of various clock genes, including Per and Cry. Ketamine has been found to time-dependently alter Clock: Bmal1-mediated transcription, leading to alteration in the expression of multiple clock genes and subsequent changes in circadian rhythm (106). (2) Light signals are conveyed to the SCN via a specialized neural pathway originating from retinal ganglion cells, known as the retinohypothalamic tract (RHT). This afferent pathway mediates the synaptic release of glutamate within the SCN. Subsequent glutamatergic signaling induces significant transcriptional activation of fundamental circadian regulators, particularly the Per1 and Per2 genes, thereby playing a critical role in regulating circadian rhythm (107). As NMDA receptor antagonists, ketamine and esketamine inhibit the transcription of these core clock genes in the SCN by blocking glutamate receptors, ultimately leading to alteration in circadian rhythm. (3) A study has found that the mTOR signaling pathway plays an essential role in the light-mediated regulation of the SCN circadian clock. mTOR affects the phase regulation of the circadian clock and the expression of core clock genes by regulating light-induced protein translation (108). As previously mentioned, ketamine and esketamine activate the mTOR pathway, suggesting that they may alter circadian rhythm by affecting mTOR signaling.

## 7 Conclusion and future research directions

The decline in postoperative sleep quality is a common complication following surgery, influenced by numerous factors, and it significantly hinders patients’ recovery. Leveraging its unique pharmacological properties, esketamine has demonstrated potential in enhancing postoperative sleep quality, it not only achieves through directly regulating the sleep-wake center circuit, but may also be related to multiple indirect mechanisms such as analgesia, anti-depression, anti-inflammation, and regulation of the circadian rhythm.

In the future, before any potential widespread clinical adoption, several key steps need to be completed. The first and most important step is to conduct large-scale, multicenter RCTs, which are crucial for clearly establishing the efficacy and safety. However, it should be emphasized that the current use of esketamine for the management of PSD is an off-label use. Therefore, clinicians must act with caution, ensure a comprehensive risk-benefit assessment when using it, and obtain full informed consent from patients regarding its experimental nature in this indication. In addition, standardized monitoring guidelines for adverse reactions need to be developed.

Such research will provide a more robust foundation for improving patients’ quality of life after surgery. Concurrently, fundamental research is imperative to delineate the precise molecular mechanisms of esketamine enhancing sleep, to explore potential therapeutic targets, and to offer theoretical support for targeted medication strategies.
