# Supplementary material for: Esketamine for postoperative sleep disturbance: clinical evidence, mechanisms, and future directions
Source: Front Psychiatry. 2025 Nov 5;16:1612230. doi: 10.3389/fpsyt.2025.1612230 (PMC12626965; doi:10.3389/fpsyt.2025.1612230)
Supplement: Supplementary file 1 [file SupplementaryFile1.docx]

**Methods**

**Search Strategy**

A structured search was carried out using the PubMed database, covering the period from its inception to December 2024, with the keywords "ketamine", "esketamine", and "sleep". The search was restricted to articles published in English.

**The search formula of PubMed**

((("Ketamine"[Mesh]) OR ((((((ketamine[Title/Abstract]) OR (Ketalar[Title/Abstract])) OR (Ketanest[Title/Abstract])) OR (Ketamine Hydrochloride[Title/Abstract])) OR (Kalipsol[Title/Abstract])) OR (Ketaset[Title/Abstract]))) OR (("Esketamine" [Supplementary Concept]) OR (((((esketamine[Title/Abstract]) OR (L-Ketamine[Title/Abstract])) OR ((-)-Ketamine[Title/Abstract])) OR (S-Ketamine[Title/Abstract])) OR (Spravato[Title/Abstract])))) AND (("Sleep"[Mesh]) OR ((((((sleep[Title/Abstract]) OR (Sleep*[Title/Abstract])) OR (Insomnia[Title/Abstract])) OR (Circadian Rhythm[Title/Abstract])) OR (Polysomnography[Title/Abstract])) OR (PSG[Title/Abstract])))

**Inclusion and Exclusion Criteria**

Inclusion criteria: Original studies (any design), reviews, and meta-analyses involving the use of ketamine/esketamine and reporting sleep-related parameters.

Exclusion criteria: Pediatric research; non-English literature; unable to obtain full text.

**Results**

Combining "ketamine" and "esketamine" with "sleep", 684 relevant articles were retrieved. After removing 5 duplicate records, 679 records remained for screening. We evaluated the titles and abstracts of these records and excluded 569 records based on the inclusion/exclusion criteria. 110 studies that might meet the criteria were initially included. We also manually searched the reference lists of relevant literature and added 3 studies. In the end, 113 studies were included in our review for reference. The detailed screening process is shown in the Fig. 1.


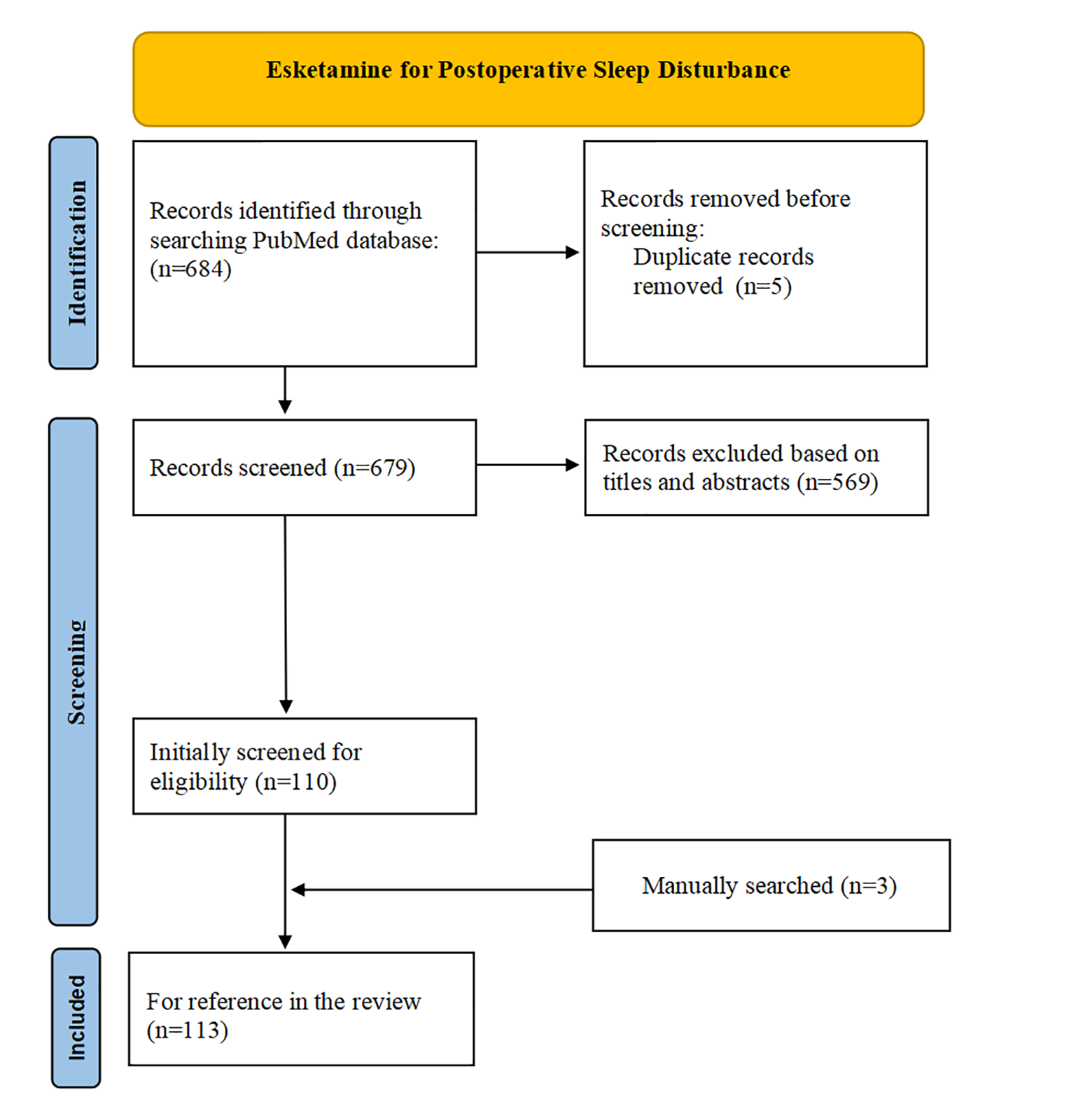


Fig. 1 The PRISMA diagram used for research screening.
